# Retinal blood flow in critical illness and systemic disease: a review

**DOI:** 10.1186/s13613-020-00768-3

**Published:** 2020-11-12

**Authors:** E. Courtie, T. Veenith, A. Logan, A. K. Denniston, R. J. Blanch

**Affiliations:** 1 Neuroscience and Ophthalmology, Institute of Inflammation and Ageing, College of Medical and Dental Sciences, University of Birmingham, Birmingham, UK; 2 Ophthalmology Department, University Hospitals Birmingham NHS Foundation Trust, Birmingham, UK; 3 NIHR Surgical Reconstruction and Microbiology Research Centre, University Hospitals Birmingham NHS Foundation Trust, Birmingham, UK; 4 Critical Care Unit, University Hospitals Birmingham NHS Foundation Trust, Birmingham, UK; 5 Birmingham Acute Care Research Group, Institute of Inflammation and Ageing, College of Medical and Dental Sciences, University of Birmingham, Birmingham, UK; 6 Axolotl Consulting Ltd, Droitwich, WR9 0JS Worcestershire UK; 7 Division of Biomedical Sciences, Warwick Medical School, University of Warwick, Coventry, CV4 7HL UK; 8 NIHR Biomedical Research Centre for Ophthalmology, Moorfields Eye Hospital NHS Foundation Trust and UCL Institute of Ophthalmology, London, UK; 9 Centre for Rare Diseases, Institute of Translational Medicine, Birmingham Health Partners, Birmingham, UK; 10 Academic Department of Military Surgery and Trauma, Royal Centre for Defence Medicine, Birmingham, UK

**Keywords:** Critical illness, Retinal blood flow, Optical coherence tomography angiography

## Abstract

**Background:**

Assessment and maintenance of end-organ perfusion are key to resuscitation in critical illness, although there are limited direct methods or proxy measures to assess cerebral perfusion. Novel non-invasive methods of monitoring microcirculation in critically ill patients offer the potential for real-time updates to improve patient outcomes.

**Main body:**

Parallel mechanisms autoregulate retinal and cerebral microcirculation to maintain blood flow to meet metabolic demands across a range of perfusion pressures. Cerebral blood flow (CBF) is reduced and autoregulation impaired in sepsis, but current methods to image CBF do not reproducibly assess the microcirculation. Peripheral microcirculatory blood flow may be imaged in sublingual and conjunctival mucosa and is impaired in sepsis. Retinal microcirculation can be directly imaged by optical coherence tomography angiography (OCTA) during perfusion-deficit states such as sepsis, and other systemic haemodynamic disturbances such as acute coronary syndrome, and systemic inflammatory conditions such as inflammatory bowel disease.

**Conclusion:**

Monitoring microcirculatory flow offers the potential to enhance monitoring in the care of critically ill patients, and imaging retinal blood flow during critical illness offers a potential biomarker for cerebral microcirculatory perfusion.

## Introduction

Critical illness with multiple organ dysfunction is characterised by complex physiological and metabolic responses requiring support and optimisation of organ systems in the intensive treatment unit (ITU) [1]. Common aetiologies include sepsis (60%), trauma, and perioperative care. Sepsis is a systemic inflammatory response to infection, mediated by the pathogen and host factors, ultimately causing multiple organ failure [2], and is a growing global concern with an estimated 48.9 million incident cases recorded worldwide in 2017, 11 million of which were fatal [3]. Septic shock describes a profound haemodynamic alteration associated with organ dysfunction, including hypovolaemia and myocardial depression [4]. Early diagnosis of sepsis and prompt treatment to reduce multiple organ failure reduces mortality [5], but survivors often have physical and neurocognitive disability referred to as post-intensive care syndrome (PICS) [6]. Attempts to improve perfusion and end-organ microcirculation using inotropes and fluids have produced variable results [7].

Microcirculation facilitates tissue oxygenation and the exchange of substances between tissues and blood. In septic shock, physiological haemodynamic parameters, such as mean arterial pressure (MAP), may not be indicative of microcirculatory perfusion [8]. Patients with sepsis often have microcirculatory alterations, such as reduced functional capillary density, which reduces oxygen delivery to vital organs and plays a key role in the development of organ dysfunction [4, 9, 10]. While the extent of these microcirculatory alterations in the brain is less well characterised than in other organs, post-mortem examination of septic patients demonstrated multiple small ischaemic lesions, suggesting microvascular insufficiency [11]. Sepsis-associated brain dysfunction (SABD) is a common sepsis-related organ dysfunction [12], and probably involves reduced cerebral blood flow (CBF) causing cerebral ischaemia [12]. Compromised cerebral blood supply often causes both immediate and delayed irreversible damage with associated neurocognitive decline and poor outcome [13]. It is, therefore, essential to be able to monitor CBF during critical illness.

The retina and brain share similar microvascular anatomy, and while direct visualisation of CBF is difficult, retinal imaging is comparatively convenient [14]. Retinal structural and blood flow changes associated with systemic and central nervous system illness are increasingly reported [15–17] with the use of ocular imaging to assess systemic disease termed “oculomics” [18]. Retinal changes may, therefore, associate with CBF in critically ill patients, offering a novel biomarker to monitor in real-time and reduce cerebral hypoperfusion.

This review discusses the relationship between cerebral and retinal blood flow, and the relevance of that relationship to systemic pathology and monitoring microcirculatory perfusion in critical illness, focussing more on sepsis.

### Cerebral and retinal blood flow autoregulation

#### Cerebral blood flow autoregulation

The human brain consumes 20% of the body’s energy at rest, dependent on CBF to ensure the delivery of oxygen, nutrients and removal of metabolic waste products [19]. Global or focal hypoperfusion rapidly results in brain damage.

Under normal physiological conditions, blood flow to the brain remains constant, in part due to the contribution of large arteries to vascular resistance, but also because of autoregulation [20]. CBF autoregulation is the ability of the brain to maintain relatively constant blood flow despite changes in perfusion pressure while matching flow to local metabolic demand [20]. Cerebral perfusion pressure (CPP) is determined by MAP and intracranial pressure (ICP), where autoregulation adjusts vascular resistance to maintain CBF. CBF autoregulation is complex, with multiple proposed overlapping regulatory mechanisms, including myogenic, neurogenic, metabolic and endothelial regulation [21]. Most data suggest reduced CBF and impaired CBF autoregulation in sepsis [22].

#### Cerebral microcirculation

The cerebral microcirculation is the driver of oxygen transport and waste removal in the cortex [23], supplied by the penetrating arteriolar network from the brain surface. Every neurone in the brain is within 20 µm of a capillary [24], receiving oxygen and nutrients yet remaining protected from fluctuations in plasma composition, circulating proteins and immune cells by the blood–brain barrier (BBB). Endothelial cells (EC) and their tight cell junctions are the fundamental constituents of the BBB and regulate paracellular transport [24].

The neurovascular unit is in part responsible for the coupling of blood flow with brain activity and is made up of EC, pericytes, astrocyte end-feet and vasoregulatory nerve terminals [25]. Pericytes project stellate, finger-like processes that ensheath the capillary wall [26] and contract or dilate in response to vasoactive mediators, such as nitric oxide (NO). NO is produced by neuronal nitric oxide synthase (nNOS) or neural pathways [27] to alter capillary diameter in autoregulation, shown in vivo in rat retina and ex vivo in cerebellar slice cultures [28]. This neurovascular coupling is impaired in the early stages of sepsis [29]. EC regulate CBF through the production of vasodilatory factors, including NO and vasoconstrictors such as endothelins, which bind to ET_A_ receptors in the cerebrovascular smooth muscle, although endothelins also have vasodilatory effects when binding to ET_B_ receptors on EC themselves [21].

#### Retinal microcirculation

**Fig. 1:**
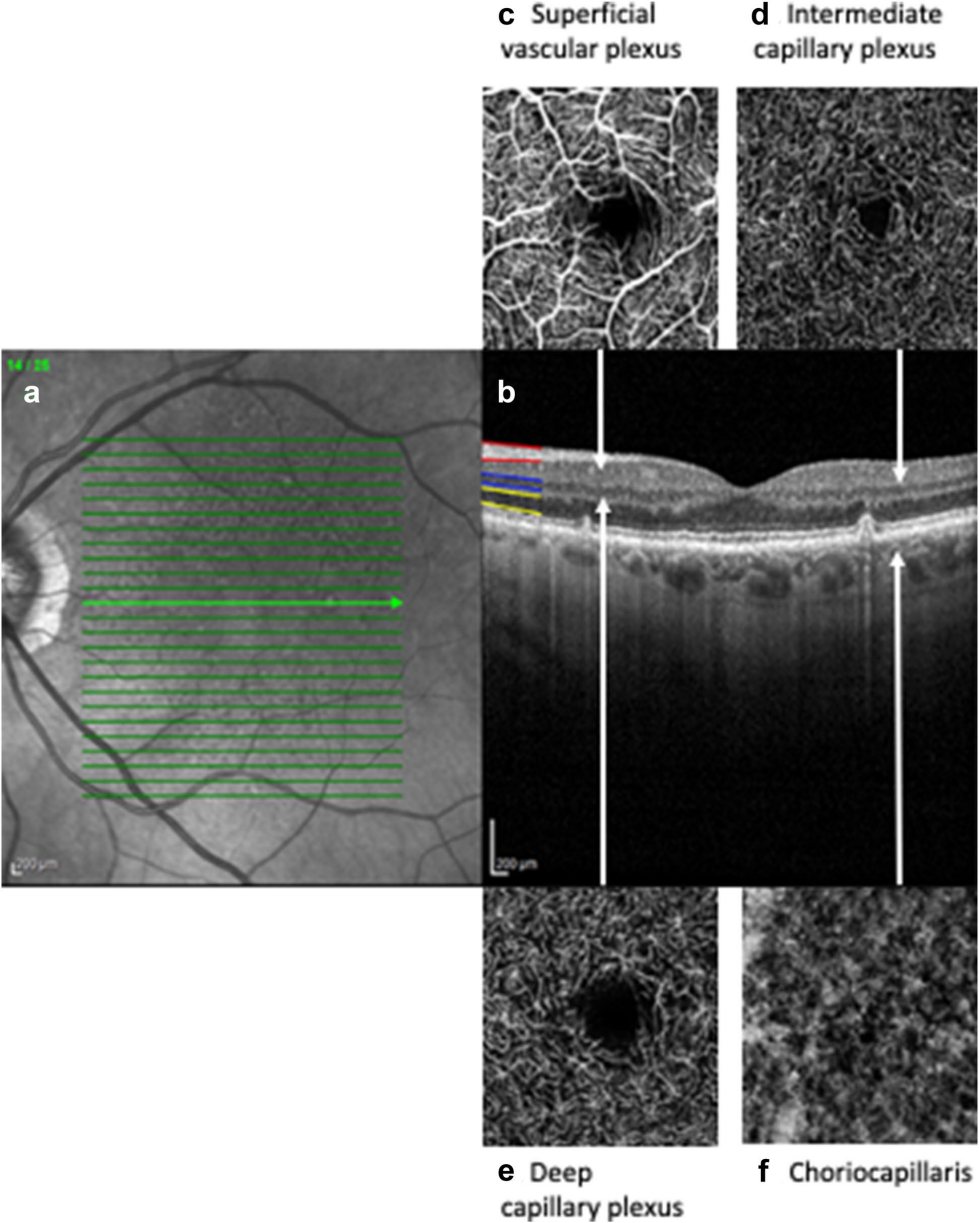
Optical coherence tomography (OCT) and optical coherence tomography angiography (OCTA) of the retina. **a** *En face* fundus image showing the optic disc and the macula*.* **b** OCT image showing the retinal layers in cross-section passing through the fovea centralis at the location indicated by the bold central arrow in (**a**). The vitreous (inside of the eye) is at the top of the image and the choroid capillary network (choriocapillaris) is at the bottom. The retinal nerve fibre layer (RNFL) is outlined in red, the inner nuclear layer in blue and the photoreceptor nuclei in yellow, using the manufacturer’s segmentation algorithm. **c** *En face* OCTA image of the superficial vascular plexus (SVP) at the level of the retinal ganglion cell nuclei (retinal level indicated by the tip of the connecting arrow). **d** *En face* OCTA image of the intermediate capillary plexus (ICP) at the inner border of the inner nuclear layer (retinal level indicated by the connecting arrow tip). **e** *En face* OCTA image of the deep capillary plexus (DCP) at the outer border of the inner nuclear layer (retinal level indicated by the connecting arrow tip). **f** *En face* OCTA image of the choriocapillaris (retinal level indicated by the connecting arrow tip)

The retinal vascular beds, derived from the central retinal artery, include the radial peripapillary capillary plexus (RPCP) in the nerve fibre layer, the superficial vascular plexus (SVP) spanning the ganglion cell layer (GCL) and inner plexiform layer, the intermediate capillary plexus (ICP) sitting between the inner plexiform layer and inner nuclear layer, and the deep capillary plexus (DCP) spanning the inner nuclear layer and outer plexiform layer [30]. These supply the inner retina, including the retinal ganglion cells, while the outer retina derives oxygenation and nutrition from the choriocapillaris of the choroid (Fig. 1) [31]. Campbell et al. propose OCTA nomenclature as the RPCP and SVP be grouped into the superficial vascular complex (SVC), with the ICP and DCP grouped into the deep vascular complex (DVC) to highlight anatomic location of the ICP at the inner plexiform/inner neuronal layer interface [30].

The foveola centralis is a depressed, avascular area of the macula, also referred to as the foveal avascular zone (FAZ). It is this area which allows the most distinct vision because of the high cone density and absence of blood vessels [31]. The circulation is particularly vulnerable in the FAZ, as the absence of retinal blood vessels leaves the cones completely dependent upon oxygen and nutrient delivery from the underlying choriocapillaris [31]. The FAZ is therefore highly sensitive to ischaemic events and because of this, can act as an indicator of several pathological processes [32]. Enlargement of the FAZ area has been associated with ischaemia in diabetic retinopathy and retinal vein occlusion [32].

The internal carotid artery gives rise to the ophthalmic artery, from which the central retinal artery arises [33], entering the optic nerve (ON) 10–12 mm behind the globe [33]. The choriocapillaris is derived from the short posterior ciliary arteries, which also branch off the ophthalmic artery. The conjunctiva covers the sclera and lines the inside of the eyelids, and is also supplied by the ophthalmic artery [34].

#### Retinal blood flow autoregulation

Similar to CBF, retinal blood flow depends on the balance between perfusion pressure in the ophthalmic artery and the resistance of the retinal vascular bed, and is autoregulated to mirror cerebral perfusion in healthy individuals [35, 36]. The retina has the highest density of microvascular pericytes in the body [36, 37], contributing to the myogenic vascular autoregulation of blood flow and providing structural support to blood vessels [36]. Changes in ocular perfusion pressure and altered metabolic demand initiate an autoregulatory response [38], maintaining retinal but not choroidal or conjunctival blood flow [35].

Retinal circulation lacks autonomic innervation [39] and is dependent on local vasogenic factors acting on the neurovascular unit [39, 40]. Despite the absence of sympathetic activation, retinal blood flow is able to remain constant over a range of intraocular pressures (IOP), which naturally fluctuates in daily life [38], although an elevated IOP above 40 mmHg reduces retinal blood flow [41]. Local metabolic factors mediating retinal autoregulation include endothelin-1 which is secreted by EC and acts as a vasoconstrictor, affecting retinal vascular endothelium, pericytes and the choroid [39]. The blood–retina barrier (BRB) has a similar structure to the BBB and protects retinal neurones from fluctuating plasma composition [42].

### Assessment of cerebral blood flow in sepsis

Functional imaging techniques to assess CBF in real-time include direct methods: triple oxygen (^15^O) positron emission tomography (^15^OPET), single-photon emission computed tomography (SPECT), and magnetic resonance angiography (MRA); and indirect methods: computed tomography perfusion (CTP), functional magnetic resonance imaging (fMRI) and near-infrared spectroscopy (NIRS) [43–45]. ^15^OPET and SPECT use isotopes which are expensive, time-consuming, and expose the patient to radiation. MRA is also expensive and time-consuming, and has poor temporal resolution. fMRI assesses regional variation in the ratio of oxy- to deoxyhaemoglobin, which associate with local changes in CBF, but is primarily sensitive to venous blood flow [44]. However, use of these imaging modalities to assess CBF in septic patients has not yet been reported. Further, these techniques require the transfer of the patient to a radiology unit [46], and the transfer of critically ill patients exposes them to increased risk of deterioration [47].

NIRS monitors cerebral cortical arterial, venous and capillary oxygenation at the capillary level, assessing fluctuations in microcirculatory CBF [45, 48]. However, there is considerable variation in vessel measurements between patients, and measurements are attenuated by pigmented hair and skin because melanin attenuates light transmission [48]. A reduction in NIRS signal in patients with sepsis in the Emergency Department was associated with mortality [49]. However, the ability to differentiate between clinical outcome groups of interest was limited by variability [49].

Transcranial Doppler ultrasound (TDU) is a non-invasive, fast, real-time technique that uses the Doppler effect to assess moving red blood cells (RBC) within the cerebral basal arteries [50], commonly the middle cerebral artery (MCA). Current clinical and research applications include: identifying the MCA and basilar artery vasospasm after subarachnoid haemorrhage, blood flow assessment in the MCA after acute ischaemic stroke, intraoperative monitoring during coronary artery bypass graft, detecting evolving hypoperfusion after traumatic brain injury (TBI), and identifying lower cerebral blood flow velocity in Alzheimer’s disease (AD) [50, 51]. TDU demonstrates altered cerebral autoregulation in 50% of the patients with sepsis and its early stage loss was associated with SABD [12, 52, 53].

Sidestream dark field (SDF) microscopy provides dynamic bed-side images of surface microcirculation. Illumination is achieved by surrounding a central light guide by concentrically placed light-emitting diodes, providing SDF illumination [54]. Light from the illuminating outer core of the SDF probe penetrates the tissue and illuminates the microcirculation by scattering [54]. SDF requires surface exposure to assess CBF, thus limiting clinical application, but an ovine model of septic shock [10] showed that the onset of septic shock was associated with decreases in cortical cerebral perfused microcirculatory vessel density, the proportion of small perfused vessels and functional capillary density, evidencing reduced microcirculatory flow. These changes were not prevented by fluid administration and were unrelated to changes in MAP and cardiac index, providing evidence of a dissociation between brain perfusion alterations and global perfusion pressure [10]. In a further study, Taccone et al. evaluated the relationship of disturbances in brain tissue oxygenation with microvascular alterations in the ovine septic model [55]. Cerebral functional capillary density and proportion of small perfused vessels significantly decreased from baseline to septic shock onset. Brain lactate:pyruvate ratio (a measure of tissue hypoxia) was increased and brain oxygen tension reduced, likely due to impaired microvascular perfusion [55].

### Assessment of the retina and retinal blood flow

#### OCT

Optical coherence tomography (OCT) allows non-contact, high-resolution cross-sectional retinal imaging [56]. A low coherence light beam is directed toward the target tissue and split into two paths [57]. While one of the light paths travels to the sample tissue—being scattered and reflected back as it passes through—the other travels to a reference mirror and is also reflected back from a known distance [58]. The two reflected light beams interact to produce interference patterns—which depend on the different path lengths—and amplitude information, which makes up the axial scan (A-scan) [59]. Multiple adjacent A-scans captured at several depths combine to produce a 2-dimensional B-scan. Adjacent B-scans form a volumetric retinal image.

Time-domain OCT (TD-OCT) was the first developed OCT which required a moving reference mirror, so had a scan rate of only 400 A-scans per second and a resolution of 8–10 µm [56].

Spectral-domain OCT (SD-OCT, a type of Fourier domain) followed, managing 20,000–130,000 A-scans per second and a resolution of 5–7 µm, by detecting multiple frequencies of light simultaneously (Optopol REVO NX OCT/OCTA, Spectrum, UK) [56].

Swept-source OCT (SS-OCT, also Fourier domain) uses a tunable laser light source, varying the emitted frequency to derive reflectivity data for each wavelength [60]. This increases signal quality in deep tissue compared to SD techniques, because of the greater penetrance of longer wavelengths.

#### Laser Doppler velocimetry

The first study attempting to quantify retinal blood flow in humans in 1985 used bidirectional laser Doppler velocimetry (LDV) [61] to measure retinal blood flow velocity and vessel diameters from fundus images, with arteriolar diameters at the site of LDV measuring between 39 and 134 µm and venules measuring from 64–177 µm. However, this and subsequent studies show high variability in mean blood flow, which is most likely explained by inter-individual variability and the fact that LDV requires good fixation by the participant for up to 45 min [61–63]. LDV would, therefore, be unsuitable for use in most clinical settings.

Doppler OCT (DOCT) gives quantitative volumetric information on blood flow in arteries and veins [64], but not the retinal microcirculation. However, there are often errors in vessel diameter extraction due to shadowing effects behind the vessel obscuring the boundary [65]. Further, eye movement alters the Doppler angle, causing artefact and limiting clinical application to date [65].

#### Fundus photography and fundus fluorescein angiography

Fundus photography is used extensively in ophthalmology, with retinal fundus colour imaging allowing retinal vascular evaluation [66], and is now possible using smartphone attachments which allows portability [67]. However, classifying arteries and veins relies on the colour and diameter of the blood vessels, which may be unreliable between images and does not directly assess microcirculation [66].

Fundus fluorescein angiography (FFA) has been used to image retinal blood flow after intravenous fluorescein injection since the 1930s [68], and images the superficial retinal vasculature, which can be obscured by leakage or haemorrhage from surrounding capillaries [69] and which itself obscures the deeper vasculature [70]. It is therefore not routinely possible to image all retinal capillary layers using FFA [70].

#### OCT angiography

OCT angiography (OCTA), developed from OCT, uses moving RBC as an intrinsic contrast medium to give 3-dimensional images of retinal and choroidal blood flow [71] without the need for injectable contrast [72]. OCTA is non-contact, non-invasive, faster and cheaper to run than FFA, with no risk of morbidity from allergic reactions to fluorescein [73], although it does not provide direct information on vascular permeability. Unlike FFA, OCTA is the result of mathematic algorithms which allow estimation of reflectivity and ultimately, for OCTA, allow blood flow detection in arteries, veins and capillaries [74, 75]. Algorithms utilise the component differences of the varying B-scans [76]. For instance, the OCT signals of SD-OCT and SS-OCT contain intensity (the strength of reflected signal) and phase (the time taken for the reflected signal to return) information; therefore algorithms may be based on intensity, phase, or both intensity and phase of OCT signals, to determine blood flow [77].

Examples of other approaches used for OCTA include: split-spectrum amplitude decorrelation angiography (analyses amplitude changes of the OCT signal, while splitting the spectrum reduces bulk-motion noise [78]); optical microangiography (includes directional information); and OCTA ratio analysis (intensity ratio calculation improves microvasculature detection sensitivity [77, 77]).

OCTA is now used alongside OCT and FFA in the diagnosis and management of numerous retinal diseases [80], including age-related macular degeneration and diabetic retinopathy [81], and in animal research [82]. Recent developments in OCT and OCTA increase portability and show feasibility for use in a critical care setting and therefore the potential to assess retinal blood flow in this group of patients [83], although the number of images may be limited within the context of usual ITU care, and by unconscious patients and semi-conscious patients who may be uncooperative and prevent imaging entirely [83]. In an ITU clinical environment, two operators are needed to acquire the scans, the devices are bulky, and given the significant cost of the device, it needs to be clearer that it provides significant value in terms of its performance, feasibility and utility in the ITU environment, including on ventilated patients.

OCTA is not without its limitations, probably the most significant being scan artefacts caused by eye movement, or projection artefacts from other retinal vessels. Further, artefacts show up differently on the scan depending on what caused them, so it is important to be able to distinguish between them. Motion artefacts from blinking show up as dark lines, while artefacts from eye movements show up as horizontal white lines [84]. These can be reduced with use of an incorporated eye-tracker, although may still increase acquisition time [84].

Projection artefacts can result when superficial blood vessels obscure deeper layers, leading to inaccurate interpretation of deeper vessel blood flow. OCTA platforms have endeavoured to reduce this by incorporating projection-masking software, but are unable to minimise projections in all layers [84].

The many different algorithms used to detect blood flow and segment retinal layers and capillary boundaries [77] make comparison of OCTA studies between devices difficult [74]. Retinal layer segmentation can also be inaccurate, which may be apparent as dark areas on the *en face* OCTA image, requiring manual adjustment prior to final interpretation [84]. It is also possible for flow to be incorrectly detected using OCTA, relating to the time difference between successive B-scans. Normal SD-OCTA has an interscan time of only 5 ms, so if the flow is too slow or fast then the B-scans would display no difference, and therefore show no flow [85].

While the image produced by an OCTA scan shows the presence or absence of blood flow, it does not give information on the speed, direction, or volume. Most commercial devices do not include automated calculation of these characteristics and the measurements are not uniform across devices which do [85], creating difficulty when comparing studies. It is therefore necessary for some studies to use third party software to quantify the data, such as measuring the FAZ area and perimeter [85], or by using either the binary or skeletonised images to calculate: perfusion density; vessel length density; and fractal dimension [86].

Finally, as OCTA is relatively new, normative data are developing [87–89], with some unknowns regarding the correlation between general parameters and vessel density [90]. There is systematic variation in FAZ area between devices (measuring higher with Heidelberg than Canon devices) but with a very high intraclass correlation coefficient (ICC) of 0.96 [91], compared to an ICC for flow index of 0.62–0.67 [92] and vessel density of 0.74–0.81 [93].

### Association of retinal and cerebral neurodegeneration

Structural retinal imaging techniques demonstrate retinal changes associated with systemic disease (Table 1) [14]. Cerebral neurodegeneration is associated with retinal neurodegeneration in acute and chronic insults, including stroke (Merge EyeScanner camera) [94], Parkinson’s disease (PD; RTVue XR Avanti SD-OCTA) [95], AD (Spectralis OCT and dynamic vessel analyser) [96, 97] and Huntington’s disease (HD; Heidelberg Spectralis OCT) [98].

**Table 1:** Studies of retinal blood flow changes in cerebral pathology

Cerebral studies	Species	Imaging modality	Pathology	Metrics measured	Findings
Frost et al. [94]	Human	Retinal photograph—Merge EyeScanner Camera	Stroke	Retinal vessels widths	Positive correlation between width of arterioles/venules and carotid disease in stroke patients
Kwapong et al. [95]	Human	OCTA—RTVue XR Avanti SD-OCT, Optovue	PD	Retinal vessel density of SRCP and DRCP Macula and RNFL thickness	Decreased retinal microvascular density, thinner macula, macular GCIP and inferior RNFL in PD
Querques et al. [97]	Human	DVA—Imedos Systems UG OCTA—Cirrus 5000 with Angioplex, Carl Zeiss Meditech OCT—SPECTRALIS HRA + OCT device, Heidelberg	MCI and AD	Retina artery and venous changes Perfusion density GCL thickness	DVA found arterial dilation decreased in the AD group compared with MCI and control groups and decreased vessel reaction in AD and MCI groups compared with control No differences in OCTA GCL thickness reduced in central and temporal sectors of AD patients compared with controls
Kwa [105]	Human	Retinal photograph—Optimed, Inc MRI—1.5 T Magnetom 63 SP/4000, Siemens AG	Cerebral SVD	Retinal arterial narrowing, crossings, sclerosis, tortuosity Presence of WML or lacunar infarcts	92% of patients with cerebral SVD had at least one retinal vascular abnormality Retinal arterial abnormalities correlated with MRI signs of cerebral SVD
Ong et al. [127]	Human	Retinal fundus photographs	Ischaemic stroke	Retinal vasculature pattern/geometry	Ischaemic stroke patients had lower fractal dimensions, greater tortuosity and narrower arteriolar calibres compared to healthy controls
Lee et al. [128]	Human	OCTA—Cirrus HD-OCT	Carotid Stenosis	Retinal vessel density of DVP	Vessel density of the DVP increased in both eyes, 1 month following treatment
Jiang et al. [102]	Human	Retinal functional imager—Optical Imaging Ltd., Israel OCT—Cirrus, Carl Zeiss Meditech, CA	MCI and AD	Retinal blood flow rate/velocity GCIPL thickness	Macular blood flow rate was significantly lower in AD patients than MCI and controls, and also significantly lower in MCI patients than controls. Blood flow velocity of arterioles was significantly lower in MCI than controls GCIPL thickness was significantly reduced in AD and MCI patients than controls
Bulut et al. [103]	Human	OCTA—RTVue XR100-2, Optovue, CA	AD	Retinal vascular density FAZ area. Choroidal thickness	AD patients had significantly lower vascular density than control group FAZ area was significantly enlarged in AD patients compared with controls Choroidal thickness was significantly lower in AD patients than controls
Zhang et al. [104]	Human	RTVue-XR OCT Avanti System—Optovue Inc, CA	Early AD and amnestic MCI	Retinal vessel/vessel length density Adjusted flow index FAZ area RNFL thickness	Patients showed a significant decrease in the parafoveal SRCP vessel density and adjusted flow index compared with controls, but not in vessel length density No difference in FAZ area between groups No significant difference in any measures in the superficial vascular complex
Lahme et al. [129]	Human	OCTA—RTVue XR Avanti with AngioVue	AD	Retinal and optic nerve head flow density	Flow density of the macula was significantly lower in AD patients than controls, found to be associated with vascular cerebral lesions in AD
Abraham et al. [130]	Human	OCTA (manufacturer not specified)	At-risk MCI	Retinal vessel density	No significant association of retinal vessel density with cognitive function or risk of MCI
Wang et al. [131]	Human	Retinal functional imaging—Optical Imaging Ltd, Israel	MS	Retinal blood flow velocity	Reduced blood flow in MS patients with and without optic neuritis compared with healthy controls
Yilmaz et al. [132]	Human	OCTA—Nidek’s RS-3000	MS	FAZ area Retinal vessel density	No difference in FAZ or perimeter, but lower macular vessel density in MS patients than healthy controls and in MS patients with optic neuritis compared to MS patients without optic neuritis
Lanzillo Cennamo, & Criscuolo [133]	Human	SD-OCT—RTVue-100 OCT, Optovue Inc OCTA—Optovue Angiovue System, Optovue Inc	MS with a history of optic neuritis	Retinal vessel density RNFL and GCL thickness	RNFL and GCL thickness and vessel density were lower in the MS group (with and without optic neuritis) than the control group
Beare and Harding [134]	Human	FFA—Topcon 50-EX, Topcon	Cerebral malaria	Retinal blood flow Tissue perfusion BRB integrity	82% of patients with cerebral malaria had perfusion abnormalities, including capillary nonperfusion, blocked retinal vessels, retinal ischaemia, intravascular filling defects and fluorescein leakage
Dallorto et al. [135]	Human	OCT and OCTA—RTVue XR Avanti	Pituitary adenoma with optic neuropathy	RNFL and ganglion cell complex thickness Vessel density	Vessel density, RNFL thickness and ganglion cell complex thickness were all decreased in pituitary adenoma patients with optic neuropathy compared to healthy eyes
Suzuki et al. [136]	Human	OCTA—DRI OCT Triton Plus	Chiasmal compression band atrophy	Vessel density RNFL	Those with band atrophy showed smaller average vessel density than controls, which had a strong correlation with RNFL thinning
Lee et al. [137]	Human	OCTA—DRI OCT Triton Plus	Pituitary tumour chiasmal compression	Vessel density RNFL and GCL thickness	Before tumour removal, vessel densities, RNFL and GCL thickness were all reduced in eyes with chiasmal compression compared with healthy controls

Summary of studies investigating retinal blood flow and microvascular changes in cerebral pathology

OCTA, optical coherence tomography angiography; OCT, optical coherence tomography; SD-OCT, spectral-domain OCT; PD, Parkinson’s disease; SRCP, superficial retinal capillary plexus; DRCP, deep retinal capillary plexus; DVP, deep vascular plexus; RNFL, retinal nerve fibre layer; GCIPL, ganglion cell layer and inner plexiform layer; DVA, dynamic vessel analyser; MCI, mild cognitive impairment; AD, Alzheimer’s disease; GCL, ganglion cell layer; MRI, magnetic resonance imaging; SVD, small-vessel disease; WML, white matter lesions; FFA, fundus fluorescein angiography; BRB, blood–retina barrier; MS, multiple sclerosis

With cerebral vasculature implicated in various neurodegenerative disorders, retinal neurodegeneration and vasculature manifestations of these disorders inform the retinal–cerebral blood flow relationship [99]. In PD, there was reduced retinal microvascular density in the superficial capillary layer of PD patients compared to healthy controls, suggesting either that PD may associate with cerebral small-vessel disease (SVD), as seen in autopsy studies, or that PD-associated retinal neurodegeneration reduces retinal blood flow [95]. In PICS associated with cognitive impairment [100], the retinal vascular changes during acute illness and afterwards may similarly mirror cerebral hypoperfusion and microvascular dysfunction.

OCT demonstrates retinal structural changes in AD patients compared with healthy individuals, including GCL loss [101] and ganglion cell inner plexiform layer loss in certain sections of the retina [102]. In patients with mild cognitive impairment (early AD) and established AD, OCTA showed lower retinal blood flow by measuring blood flow rate and blood flow velocity in both retinal arteries and veins, showing lower vascular density in the macular, foveal and parafoveal zones and larger FAZ areas compared to cognitively normal patients [102–104].

In a study investigating the relationship between retinal arterial disease and cerebral SVD, 60% of patients with a systemic atherosclerotic disease showed signs of cerebral SVD on MRI [105]. 92% of these individuals had at least one retinal arterial abnormality irrespective of the presence of hypertension, suggesting that retinal signs are more sensitive than SVD on cerebral MRI in detecting cerebrovascular disease [105].

Cerebral neurodegenerative disorders cause retinal structural changes and secondary retinal blood flow changes, whilst cerebrovascular disease also reduces retinal perfusion, providing evidence that pathological changes to cerebral perfusion and cerebral neurodegeneration both affect retinal perfusion. This is particularly relevant to critical illness in which cerebral hypoperfusion or hyperperfusion may be both caused by and contribute to cerebral dysfunction and damage, and altered retinal blood flow may relate to both systemic hypoperfusion and sepsis-induced neurodegeneration [22].

### Conjunctival and sublingual microcirculation in sepsis

Techniques to monitor surface microcirculatory changes directly include: SDF videomicroscopy which developed from orthogonal polarisation spectral (OPS); incident dark field (IDF) imaging; laser Doppler perfusion imaging (LDPI); and laser speckle contrast imaging (LSCI) [106]. OPS demonstrates reduced sublingual microvascular blood flow in patients with severe sepsis by direct visualisation, and correlated microvascular alterations with survival of septic patients [107], while SDF demonstrates hypoperfusion and increased heterogeneity in septic microcirculation [108]. The sublingual area is the site used most to evaluate microcirculation in critically ill patients, with SDF the current standard method to do this [109]. With the introduction of handheld video microscopes, SDF also allows bedside monitoring of microcirculation, but it is not yet widely used in clinical practice [8]. A major drawback of SDF is that it can only monitor skin and mucosal blood flow and requires direct contact with the skin, causing pressure and motion artefacts, posing technical challenges which reduces video quality and reliability [110].

IDF uses a green light source that is absorbed by haemoglobin to detect RBC [34] with devices optimised for surface microcirculatory visualisation and may have better image quality than SDF imaging [111]. Portable IDF (Cytocam®-IDF device) demonstrated reductions in all microcirculatory parameters of the conjunctiva, including microvascular flow index (MFI) and total and perfused vessel density, in septic patients compared with healthy individuals [34]. Similarly, in the ovine septic and haemorrhagic shock model, functional capillary density and MFI of the conjunctiva capillary microcirculation were significantly reduced in septic shock, with alterations correlating with sublingual capillary microcirculation [112]. SDF in a pig sepsis model (Microscan; Microvision Medical) showed significant decreases in MFI and proportion of perfused small vessels (venules and capillaries with diameters < 20 µm) in the conjunctival, sublingual, jejunal and rectal mucosal microcirculation following sepsis onset [113].

LDPI and LSCI are non-contact techniques, but measure average Doppler shift and therefore only assess relative flow changes, normalised to baseline values [106]. In contrast, OCTA requires no contact and has recently shown suitability for evaluating sublingual microcirculation in healthy volunteers, suggesting it is a promising method for peripheral (as well as retinal) microcirculatory evaluation [8].

### Retinal blood flow changes associated with systemic pathology

#### Retinal blood flow in cardiovascular and inflammatory disease

Retinal changes in malignant hypertension are well-recognised. When patients with malignant hypertension were compared to controls, some, but not all, measures of vessel density and skeletal density of the superficial retinal layer and deep retinal layer were reduced in the hypertensive group, demonstrating retinal capillary dropout associated with malignant hypertension using OCTA [114]. Retinal capillary density was reduced in the DVP of patients with poorly controlled blood pressure compared with those with well controlled blood pressure, further highlighting the potential role of OCTA to monitor early microvascular changes arising from systemic hypertension [15]. Further studies would answer the extent of which these changes are associated with microvascular complications and end-organ damage [114].

Patients with atrial fibrillation have abnormal retinal electrophysiological responses and lower flow density in the macular and ON SVP on OCTA compared with healthy controls, which partially normalised when patients were restored to sinus rhythm, but showed no evidence of a difference in FAZ area [115].

Patients suffering acute coronary syndromes also have abnormal retinal blood flow on OCTA, with the lowest inner retinal vessel density in the highest risk patients (highest American Heart Association scores and the lowest left ventricular ejection fractions) [116]. Some early-stage coronary heart disease patients could be defined as a high-risk population on OCTA by reduced retinal vessel density, and reduced choroidal vessel density and blood flow, suggesting an efficient and non-invasive method for detection of early-stage coronary heart disease [117]. Taken together, the findings in cardiac disease suggest that impaired cardiac output reduces retinal blood flow, especially given the partial normalisation when sinus rhythm is restored. However, the previous studies demonstrating preserved, autoregulated retinal blood flow under hypovolaemic stress also suggest that, common to the studies of systemic and cerebrovascular disease, at least some of the OCTA abnormalities observed reflect a long-term vasculopathy.

There was no association between a diagnosis of Crohn’s disease or ulcerative colitis and retinal blood flow, but when either group of patients had active disease, FAZ area was reduced compared to patients in remission, suggesting altered retinal blood flow autoregulation by systemic inflammatory status [16]. Systemic sclerosis has involvement of the microvasculature as one of the earliest features. OCTA showed significantly decreased foveal, parafoveal and perifoveal vessel densities in the superficial capillary plexus, and foveal vessel density in the DCP, of patients with systemic sclerosis compared with healthy individuals [118]. These results suggest indicators of retinal vascular injury before patients become symptomatic [118].

Pregnancy is a state with hyperdynamic circulation and a finely modulated immune system [119]. Pre-eclampsia is associated with generalised endothelial dysfunction, increasing vascular resistance and leakage from blood vessels and manifesting as hypertension, proteinuria and oedema, but no microcirculatory changes detectable by SDF [17]. In contrast, patients in the third trimester of pregnancy have reduced macular SVP vessel density. Macular SVP and ICP vessel density in high-risk pregnancies are also lower than in low-risk pregnancies, and patients with pre-eclampsia also have reduced macular SVP and ICP, but increased peripapillary SVP perfusion compared to patients with uncomplicated pregnancy and normal controls [120].

A prototype handheld SS-OCTA device was used to capture high-quality vitreoretinal images in awake premature neonates at risk for retinopathy of prematurity, with greater imaging speed and detail compared with currently available handheld SD-OCT devices [121].

### Retinal microcirculation in sepsis and haemorrhagic shock

In a pig model of acute respiratory distress syndrome [122], RNFL thickness was increased and there was immunostaining for reactive oxygen species HIF-1α and VEGF-A in retinal arterioles, suggestive of increased retinal vascular permeability and endothelial dysfunction [122].

After ovine haemorrhagic shock [123], SVP flow density on OCTA decreased from 44.7% baseline to 34.5%, recovering to 46.9% after fluid resuscitation, correlating with systemic haemodynamic parameters. Conjunctival microcirculation assessed using IDF microscopy also showed a reduced proportion of perfused vessels from 100% to 72%, which returned to 98.7% after resuscitation [123]. The alterations in OCTA flow density correlated with reduced perfused vessel density in IDF of the conjunctiva and haemodynamic parameters (MAP, heart rate and cardiac index all decreased), suggesting that both the retinal and conjunctival microcirculatory changes may relate to cerebral perfusion alterations. In contrast, in a rat haemorrhagic shock model, choroidal blood flow dropped in proportion to MAP (preceding increases in serum lactate), but retinal blood flow assessed by OCTA was maintained [124].

FFA in patients with sepsis demonstrated prolonged retinal arterial filling time after intravenous dye injection, associated with fundus signs of retinal vasculopathy including haemorrhages and microaneurysms, although retinal arteriolar diameters were not measured [125]. Patients with delayed retinal arterial filling had a lower cardiac index, higher Acute Physiology and Chronic Health Evaluation II scores and lower interleukin-6 and C-reactive protein levels, suggesting an impaired inflammatory response [125].

Septic patients in the ITU had increased average retinal arteriolar calibres (165 µm[149–187 µm] vs. 146 µm[142–158 µm], *p* = 0.002) compared with healthy controls and decreased vascular length density (0.51% vs. 0.64%, *p* < 0.001) on portable fundus photography compared with healthy controls [126].

There is a need for improved monitoring of cerebral perfusion in a critical care environment to allow perfusion-directed resuscitation, improve patient outcomes, and possibly reduce long-term cognitive impairment. These retinal imaging studies demonstrate that retinal vessel density and retinal perfusion are affected by systemic haemodynamic changes [116], and the systemic inflammatory response [16, 118], but also that it does not simply provide a mirror to systemic haemodynamic status, being resistant to change in some models [124] and providing additional information in others (Table 2) [117, 120, 125].

**Table 2:** Studies of retinal and cerebral blood flow changes in sepsis, other haemodynamic disturbances, and other inflammatory disorders

Sepsis studies	Species	Imaging modality	Pathology	Metrics measured	Findings
Taccone et al. [10]	Animal (ovine)	SDF videomicroscopy of the cerebral cortex—Microscan; MicroVision Medical	Septic model	Total perfused vessel density Functional capillary density Small perfused vessels Perfused capillaries	Septic animals showed progressive reduction in all metrics compared to control group, suggesting cerebral microcirculatory impairment
Crippa et al. [12]	Human	Transcranial Doppler of the middle cerebral artery—Compumedics DWL, Dresden, Germany	SABD	Cerebral autoregulation by blood flow velocity and mean flow index	Cerebral autoregulation impaired, cerebral blood flow increased and SABD in at least 50% of septic patients No difference in mean flow index between survivors and non-survivors
Simkiene et al. [34]	Human	Portable IDF of conjunctival microcirculation—Cytocam®, Braedius Medical, Huizen, The Netherlands Fundus imaging—Optomed Aurora (Optomed Oy, Oulu, Finland)	Sepsis or septic shock	MFI TVD PVD Retinal arteriolar and venular calibre	Lower MFI, TVD and PVD in all septic patients than healthy controls Lower MFI in non-survivors than survivors TVD and PVD and arteriolar calibre associated
De Backer and Creteur [107]	Human	OPS imaging of sublingual mucosa—Cytoscan ARII; Cytometrics	Sepsis, pre-cardiac surgery patients, ICU controls	Vascular density	Septic patients had decreased vascular density compared with all other groups
Kanoore Edul et al. [108]	Human	SDF microscopy imaging of sublingual mucosa—Microscan; MicroVision Medical	Septic shock	MFI Perfused capillary density	Hypoperfusion and microcirculatory abnormalities in septic patients compared with healthy controls, which were more severe in non-survivors
Hessler et al. [112]	Ovine	Portable IDF of sublingual and conjunctival microcirculation—Cytocam®, Braedius Medical, Huizen, The Netherlands	Sepsis and haemorrhagic shock	MFI TVD PVD PPV	Lower PVD, PPV and MFI in haemorrhagic and septic shock in mouth and conjunctiva than control group No change in TVD
Pranskunas et al. [113]	Pig	SDF videomicroscopy of sublingual, conjunctival, jejunal and rectal microcirculation—Microscan; Microvision Medical, Amsterdam, The Netherlands	Sepsis	MFI PPV PVD TVD	Lower MFI and PPV observed in all locations in the sepsis group than control group, with the lowest MFI in sublingual region No change in TVD Sublingual associates with conjunctival MFI
Taccone et al. [55]	Animal (ovine)	SDF videomicroscopy of cerebral cortex—MicroScan (MicroVisionMedical, Amsterdam, The Netherlands)	Sepsis	Mean flow index Proportion of small perfused vessels Functional capillary density Lactate/pyruvate ratio (tissue hypoxia)	Cerebral functional capillary density and proportion of small perfused vessels decreased significantly from baseline to shock onset, compared with control group Brain oxygen levels reduced while lactate/pyruvate ratio increased in septic animals Suggests impaired cerebral microcirculation in septic animals
Alnawaiseh et al. [123]	Animal (ovine)	OCTA—RTVue XR Avanti with Angiovue, Optovue Inc	Haemorrhagic shock	Flow density	Reduced flow density of the superficial retina after shock induction, which recovered after resuscitation
Park [124]	Animal (rat)	OCTA (manufacturer not specified)	Haemorrhagic shock and sepsis	BFI against MAP and lactate	As MAP decreased in the haemorrhagic shock model, BFI of the choroid but not retinal artery decreased and was associated with increased lactate concentration in sepsis
Erikson et al. [125]	Human	FFA—HRA 2–00153; Heidelberg Engineering, Heidelberg	Sepsis	RAFT Inflammatory markers	Retinal abnormalities observed in half of the patients with sepsis, being more common in those showing PRAFT Patients with PRAFT had lower levels of CRP and IL-6 compared with patients who had SRAFT, suggesting impaired inflammatory response
Simkiene et al. [126]	Human	Fundus imaging—Optomed Aurora (Optomed Oy, Finland)	Sepsis	Retinal arteriolar and venular calibre Retinal vascular length density	Retinal arteriolar calibre higher in septic than healthy control patients, but venular calibre did not differ Septic patients had lower retinal vascular length density

Other haemodynamic disturbances studies	Species	Imaging modality	Pathology	Metrics measured	Findings
Chua et al. [15]	Human	OCTA—AngioVue; Optovue, Inc	Systemic hypertension	Retinal vessel density	Vessel density was reduced in patients with poorly controlled blood pressure compared with those with well controlled blood pressure
Terheyden [114]	Human	OCTA—PLEX Elite 9000, Carl Zeiss Meditec Inc	Systemic hypertensive crisis	Retinal vessel density Skeletal density Vessel density index	Reduced skeletal density in the S-RL and D-RL and reduced vessel density in the D-RL in hypertensive patients compared with healthy controls
Alnawaiseh [115]	Human	OCTA—RTVue XR Avanti with AngioVue, Optovue, Inc	AF	FAZ area Flow density	No difference found in FAZ area between groups Flow density in the superficial retina and ONH were lower in the AF group
Arnould and Guenancia [116]	Human/	OCTA—Cirrus HD-OCT, model 5000, Carl Zeiss Meditec	ACS	Retinal vascular density of SCP	Vascular density of SCP negatively associated with cardiovascular risk factors and left ventricular ejection fraction at admission
Wang et al. [117]	Human	OCTA—AngioVue, Optovue, Inc	Coronary heart disease	Retinal vessel density Flow area	Some early stage coronary heart disease patients showed reduced vessel density, and reduced choroidal vessel density and blood flow
Lin [120]	Human	OCTA (manufacturer not specified)	High-risk pregnancies	Retinal vessel perfusion density/vessel length density of SVP, ICP and DCP	Reduced SVP perfusion density and vessel length density in high-risk pregnancy individuals compared with low-risk pregnancy individuals

Other inflammatory disorders studies	Species	Imaging modality	Pathology	Metrics measured	Findings
Kılınç Hekimsoy et al. [118]	Human	OCTA—XR Avanti AngioVue	Systemic sclerosis	FAZ area and perimeter Vessel density	Patients with systemic sclerosis showed significantly decreased vessel density compared with controls, but there was no difference in FAZ area or perimeter
Nakayama [16]	Human	OCT/OCTA—Topcon Triton, Topcon	IBD	FAZ area	Patients with active disease showed decreased FAZ area compared with patients in remission

Summary of studies investigating retinal and cerebral blood flow and microvascular changes in sepsis, other haemodynamic disturbances and other inflammatory disorders.

SDF, sidestream dark field; SABD, sepsis-associated brain dysfunction; ICU, intensive care unit; OPS, orthogonal polarisation spectral; ICU, intensive care unit; OCTA, optical coherence tomography angiography; S-RL, superficial retinal layer; D-RL, deep retinal layer; AF, atrial fibrillation; FAZ, foveal avascular zone; IDF, incident dark field; MFI, microvascular flow index; TVD, total vessel density; PVD, perfused vessel density; PPV, proportion of perfused vessels; ONH, optic nerve head; ACS, acute coronary syndrome; SCP, superficial capillary plexus; OCT, optical coherence tomography; IBD, inflammatory bowel disease; SVP, superficial vascular plexus; ICP, intermediate capillary plexus; DCP, deep capillary plexus; BFI, blood flow index; MAP, mean arterial pressure; FFA, fundus fluorescein angiography; RAFT, retinal arterial filling time; PRAFT, prolonged RAFT; SRAFT, short RAFT

## Conclusions

The reviewed studies demonstrate the link between retinal and cerebral blood flow, and that changes in retinal perfusion reflect changes in cerebral microcirculation. Retinal blood flow is altered by systemic and microcirculatory hypoperfusion, and is in association with cerebral and retinal neurodegeneration. Conjunctival and sublingual microcirculation are also altered in sepsis. Of the different retinal blood flow imaging modalities, OCTA is the least invasive and is a promising method for retinal evaluation in the future. Retinal blood flow, therefore, has potential as a biomarker of systemic disease, with developing evidence in critical illness and sepsis.

## Data Availability

Not applicable.

## Abbreviations

ITU: Intensive treatment unit
PICS: Post-intensive care syndrome
MAP: Mean arterial pressure
SABD: Sepsis-associated brain dysfunction
CBF: Cerebral blood flow
CPP: Cerebral perfusion pressure
ICP: Intracranial pressure
BBB: Blood–brain barrier
EC: Endothelial cells
NO: Nitric oxide
nNOS: Neuronal nitric oxide synthase
RPCP: Radial peripapillary capillary plexus
SVP: Superficial vascular plexus
GCL: Ganglion cell layer
ICP: Intermediate capillary plexus
DCP: Deep capillary plexus
SVC: Superficial vascular complex
DVC: Deep vascular complex
FAZ: Foveal avascular zone
RNFL: Retinal nerve fibre layer
ON: Optic nerve
IOP: Intraocular pressure
BRB: Blood–retina barrier
CTP: Computed tomography perfusion
^15^O: Triple oxygen
^15^OPET: Triple oxygen positron emission tomography
SPECT: Single-photon emission computed tomography
MRA: Magnetic resonance angiography
NIRS: Near-infrared spectroscopy
fMRI: Functional magnetic resonance imaging
TDU: Transcranial Doppler ultrasound
RBC: Red blood cells
MCA: Middle cerebral artery
TBI: Traumatic brain injury
AD: Alzheimer’s disease
OCT: Optical coherence tomography
A-scan: Axial scan
TD-OCT: Time-domain optical coherence tomography
SD-OCT: Spectral-domain optical coherence tomography
SS-OCT: Swept-source optical coherence tomography
LDV: Laser doppler velocimetry
DOCT: Doppler optical coherence tomography
FFA: Fundus fluorescein angiography
OCTA: Optical coherence tomography angiography
ICC: Intraclass correlation coefficient
PD: Parkinson’s disease
HD: Huntington’s disease
SVD: Small vessel disease
SDF: Sidestream dark field
OPS: Orthogonal polarisation spectral
IDF: Incident dark field
LDPI: Laser Doppler perfusion imaging; LSCI
